# Morphometric Evaluation of Anterior Cruciate Ligament Orientation and Tibial Footprint Location Using Magnetic Resonance Imaging

**DOI:** 10.3390/diagnostics16050748

**Published:** 2026-03-02

**Authors:** Esra Babaoğlu, Belgin Bamaç, Kaya Memişoğlu

**Affiliations:** 1 Department of Anatomy, Faculty of Medicine, Zonguldak Bülent Ecevit University, 67100 Zonguldak, Türkiye; 2 Department of Anatomy, Faculty of Medicine, Kocaeli University, 41001 Kocaeli, Türkiye; bbamac@hotmail.com; 3 Department of Orthopedics and Traumatology, Faculty of Medicine, Kocaeli University, 41001 Kocaeli, Türkiye; kayamemisoglu@yahoo.com

**Keywords:** anterior cruciate ligament, magnetic resonance imaging, tibial footprint, knee morphometry, sagittal plane, coronal plane, sex differences, tunnel placement

## Abstract

**Background/Objectives**: The anterior cruciate ligament (ACL) plays a key role in knee stability, biomechanics, and proprioception, and is one of the most frequently injured and reconstructed ligaments in both athletes and the general population. The anatomical placement of femoral and tibial tunnels close to the native ACL insertion sites is critical for long-term clinical outcomes and graft survival. This study aimed to define sagittal and coronal ACL alignment and tibial footprint morphology on magnetic resonance imaging (MRI) in healthy knees, to explore sex- and side-related differences, and to provide population-specific reference values. **Methods**: In this retrospective cross-sectional study, knee MRIs acquired between 2018 and 2021 were screened, and knees with an intact ACL and without deformity or joint pathology that could alter alignment were included. After applying inclusion and exclusion criteria, 636 knees (320 right, 316 left) from 545 individuals (338 women, 298 men; 15–80 years, mean age 34.87 ± 11.65 years) were analyzed. On sagittal images, the sagittal ACL angle (S-ANGLE) was measured on the slice where the ligament appeared maximally visualized. The midpoints of the ACL were identified on two adjacent sagittal slices, and a line drawn through these midpoints was used to represent the central axis of the ligament; the angle between this line and the tibial plateau was recorded as the S-ANGLE. For anteroposterior localization of the tibial footprint, an anteroposterior reference distance (S-long) was defined as the length measured parallel to the tibial plateau, extending from the midpoint of the tibial tuberosity (corresponding to the insertion site of the patellar ligament and used as a topographic anterior landmark) toward the posterior aspect of the proximal tibia. A perpendicular line was drawn from the anterior end of S-long to establish the anterior reference boundary. The distance from this anterior reference line to the midpoint of the ACL tibial footprint along the same anteroposterior axis was defined as S-short. The sagittal footprint percentage (S-PERCENTAGE) was calculated as (S-short/S-long) × 100, representing the size-normalized sagittal anteroposterior position of the ACL tibial footprint midpoint. On coronal images, the ACL–tibial plateau angle (C-ANGLE), mediolateral tibial length (C-LONG), and distance from the medial edge to the ACL insertion (C-short) were obtained; C-PERCENTAGE was calculated analogously. Medial mechanical proximal tibial angle (mMPTA) was used to confirm physiological coronal alignment. Non-parametric tests were applied, with *p* < 0.05 considered statistically significant. **Results**: Women had significantly greater sagittal ACL angles than men, whereas anteroposterior distances measured from the midpoint of the tibial tuberosity (used as an anterior topographic landmark) and oriented parallel to the tibial plateau (S-LONG) and mediolateral tibial lengths (C-LONG) and absolute distances to the ACL tibial footprint were larger in men. In contrast, normalized sagittal and coronal footprint percentages (S-PERCENTAGE, C-PERCENTAGE) did not differ meaningfully between sexes, indicating the preservation of the relative ACL tibial insertion site despite size differences. Small but statistically significant side-to-side differences were observed in some coronal parameters; however, absolute differences were small and did not substantially modify the overall alignment pattern. **Conclusions**: This study provides large-sample, population-specific reference values for ACL orientation and tibial footprint location in both sagittal and coronal planes in healthy knees. The combination of higher sagittal ACL angles and shorter anteroposterior distances reference measured from the midpoint of the tibial tuberosity and oriented parallel to the tibial plateau (S-LONG) in women may represent a structural substrate contributing to the higher ACL injury rates reported in females. The morphometric data presented here may assist in individualized ACL reconstruction planning, MRI-based assessment of tibial tunnel position, and the design of knee-related biomedical implants and devices.

## 1. Introduction

The knee joint begins to develop from a mesenchymal condensation at approximately the 4th week of gestation and becomes anatomically distinguishable by the 6th week. The anterior cruciate ligament (ACL) appears as a mesenchymal condensation within the fetal blastema around the 6th–7th gestational weeks and migrates posteriorly in parallel with the development of the intercondylar notch, while the posterior cruciate ligament (PCL) largely maintains its initial position. Both cruciate ligaments are intracapsular but extrasynovial structures [1,2,3,4].

Functionally, the ACL is taut in extension and lax in flexion, and its primary role is to limit anterior translation of the tibia relative to the femur, resisting up to approximately 86% of anteriorly directed forces [2,5,6]. It also contributes to the control of internal tibial rotation near full extension, while having a more limited effect on external rotation and varus–valgus angulation under load. In addition to its mechanical role, the ACL is characterized by a rich supply of sensory receptors, the presence of type II collagen despite the absence of hyaline cartilage, and a higher glycosaminoglycan content than tendons [2,7,8,9,10]. This specific structural organization provides resistance to tension and compression, shock absorption, rotational stability, and an important contribution to knee joint proprioception [11,12,13].

ACL injuries are among the most common ligamentous lesions of the knee, particularly in pivoting sports, and the ACL is one of the most frequently reconstructed ligaments worldwide. These injuries are associated with early functional impairment and, in the long term, with degenerative changes such as cartilage damage and osteoarthritis [6,14]. Numerous studies have demonstrated that positioning the femoral and tibial tunnels as anatomically as possible with respect to the native ACL insertion sites is associated with improved clinical and functional outcomes. Tunnel placement, graft choice, initial tension, fixation method, graft–tunnel motion, and biological healing are all critical determinants of reconstruction success. In particular, an appropriate alignment of the tibial tunnel with the native ACL orientation may reduce tunnel widening during knee flexion and thereby enhance surgical outcomes [15,16].

In this context, accurate knowledge of the ACL tibial footprint and its orientation relative to the tibial plateau in different planes is clinically relevant. MRI-based morphometric data derived from individuals without structural knee pathology can provide reference values for preoperative planning, enable the assessment of tibial tunnel placement, and aid in the development of patient-specific or population-specific implant designs. However, there is a relative paucity of MRI studies that simultaneously evaluate sagittal and coronal ACL angles and size-normalized anteroposterior and mediolateral position of the tibial footprint using standardized reference-axis-based measurements in large samples across a broad age range, while also considering sex- and side-related differences, particularly in our population.

Therefore, the aim of the present study was to use magnetic resonance imaging (MRI) to characterize the morphometric properties of the ACL tibial insertion, its sagittal and coronal orientation with respect to the tibial plateau and its size-normalized anteroposterior and mediolateral footprint position based on standardized reference-axis measurements in healthy adolescent and adult individuals. By providing population-specific reference values stratified by sex and side, we sought to contribute baseline data for future anatomical, radiological, and surgical studies related to the ACL.

## 2. Materials and Methods

### 2.1. Study Design and Population

Ethical approval required for the study was obtained from the Kocaeli University Non-Interventional Clinical Research Ethics Committee on 12 October 2021, with the number GOKAEK-2021/13.09. The study was performed according to Helsinki Declaration. This retrospective cross-sectional study was conducted at the Department of Radiology, Kocaeli University School of Medicine. All knee magnetic resonance imaging (MRI) examinations performed between 2018 and 2021 were screened. In the initial search, 2565 knee MRI studies from 1687 patients were identified. After a preliminary review based on image quality and study criteria, 666 knee MRIs from 565 patients were considered suitable for morphometric evaluation.

During the measurement phase, 6 MRI series were excluded due to inadequate technical quality, and 25 knees were excluded because of marked varus–valgus deformity or insufficient visualization for reliable angle measurements. Consequently, 636 knee MRIs from 545 patients were included in the final analysis. Individuals aged 15–80 years were eligible for inclusion, and the overall cohort had a mean age of 34.87 ± 11.65 years (median 33.0; 25th–75th percentile 26.0–42.0).

When both knees of the same individual fulfilled all eligibility criteria independently, each knee was treated as a separate observation. For descriptive purposes, participants were grouped into four age categories based on the literature: 15–18 years (late adolescence) [17], 19–39 years [18,19], 40–59 years [18,19,20], and ≥60 years [20,21]. These age groups were used solely to characterize the demographic distribution; due to the limited sample size in the extreme age groups, no formal hypothesis testing was performed across age categories.

Although age groups were defined to characterize the demographic distribution of the cohort, age was not included as a primary analytical variable. The marked imbalance in subgroup sizes, particularly in the youngest and oldest categories, was considered insufficient for statistically robust comparisons and could potentially introduce unstable estimates. Therefore, age stratification was retained for descriptive purposes only, consistent with the primary aim of the study, which focused on sex- and side-related morphometric variation.

### 2.2. Inclusion and Exclusion Criteria

Patients were eligible for inclusion if they were between 15 and 80 years of age, had knee MRI examinations with sufficient visualization of the anterior cruciate ligament (ACL) and proximal tibial structures to allow reliable angle and distance measurements, and had complete demographic information available for the imaging date.

Exclusion criteria comprised congenital or acquired lower extremity malalignment such as marked varus or valgus deformity or developmental hip dysplasia; a history of surgery for femoral or tibial fractures; early or advanced radiological signs of knee osteoarthritis; degeneration, partial or complete tear, graft, or prior reconstruction involving the ACL or posterior cruciate ligament (PCL); meniscal degeneration or tears corresponding to grade 3–4 signal alterations on MRI; collateral ligament tears; intra-articular osteochondral lesions, bone marrow edema, osteonecrosis, or structural pathology of the tibial plateau; tumoral lesions around the knee, significant synovial hypertrophy, or substantial joint effusion; and radiological features of degenerative or inflammatory arthritis associated with joint destruction. Any subject meeting at least one of these criteria was excluded from the study.

### 2.3. MRI Acquisition Protocol

All MRI examinations were performed using three different systems: a Philips Achieva 1.5 T scanner (software R3.2.3.5), a Philips Achieva dStream 3 T scanner (Philips Healthcare, Best, The Netherlands), and a GE Healthcare system (model no. 90–120, GE HealthCare Technologies, Chicago, IL, USA). Images were reviewed on the Kocaeli University picture archiving and communication system (PACS) using Sectra Workstation IDS7 (version 23.2.6.5161).

During image acquisition, patients were positioned in a feet-first supine (FFS) position with the knee placed in the maximum degree of flexion they could comfortably tolerate. The joint was stabilized as much as possible to minimize motion artifacts. Images were acquired in orthogonal planes using a slice thickness of 3 mm and an interslice distance of approximately 3.3 mm. Morphometric measurements were performed exclusively on sagittal and coronal proton density-weighted fat-suppressed (PDW-SPAIR) sequences.

Across scanner platforms, acquisition parameters were kept comparable, including repetition time (TR approximately 2800–3000 ms), echo time (TE approximately 30 ms), field of view (FOV 130–160 mm), and the use of dedicated multi-channel knee coils with 8 or 16 receiver channels, depending on the scanner platform. Only sequences meeting predefined image quality and visualization criteria for the ACL and proximal tibial anatomy were included in the analysis.

Because this was a retrospective study based on routine clinical MRI datasets, the exact knee flexion angle was not systematically recorded and therefore could not be incorporated as a covariate. To minimize potential variability related to scanner type or knee positioning, measurements were performed using predefined anatomical reference definitions, standardized slice-selection criteria, and a uniform PACS-based measurement workflow. Observer calibration sessions followed by interobserver and intraobserver reliability analyses demonstrated high ICC values, supporting the robustness and reproducibility of the measurement protocol across different imaging platforms.

### 2.4. Measurement Definitions

All morphometric and angular measurements were performed on sagittal and coronal proton density-weighted images.

**Figure 1:**
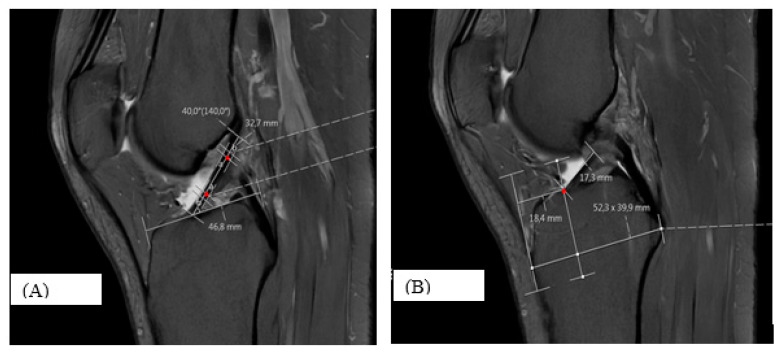
Sagittal MRI measurements of the ACL tibial insertion.

In the sagittal plane, the ACL sagittal angle (S-ANGLE) was measured on the slice where the ligament appeared thickest and most clearly delineated. Two central points were identified along the mid-axis of the ACL on adjacent slices, and the line connecting these points was used to define the ligament axis. The angle between this axis and the tibial plateau was recorded as the S-ANGLE [15] (Figure 1A).

For the sagittal tibial footprint percentage (S-PERCENT), the first sagittal slice in which the tibial insertion of the ACL became visible was identified. On this slice, the distance from the midpoint of the ACL tibial footprint to an anterior tibial reference line (defined as a line drawn perpendicular to the tibial plateau through the anterior margin of the proximal tibia) was measured as S-SHORT, and the anteroposterior reference distance measured from the midpoint of the tibial tuberosity and oriented parallel to the tibial plateau was defined as S-LONG.

Perpendicular projection lines were drawn to ensure that both distances were measured along parallel axes. S-PERCENT was calculated as the ratio S-SHORT/S-LONG and expressed as a percentage, representing the size-normalized sagittal anteroposterior position of the ACL tibial footprint midpoint [15] (Figure 1B).

(A) Definition of the sagittal ACL angle (S-ANGLE). The ligament axis was defined by connecting two central points identified along the mid-axis of the ACL on adjacent sagittal slices where the ligament appeared thickest and most clearly delineated. The angle between this axis and the tibial plateau was recorded as the S-ANGLE. Red dots indicate the midpoint markers used to define the ACL mid-axis. Measurement lines and angular values represent PACS-generated overlays. (B) Measurement of the sagittal tibial footprint percentage (S-PERCENT) calculated as the ratio of the distance from the midpoint of the ACL tibial footprint to the anterior tibial reference line (S-SHORT) to the anteroposterior reference distance measured from the midpoint of the tibial tuberosity and oriented parallel to the tibial plateau (S-LONG). The red dot indicates the midpoint of the ACL tibial footprint on the sagittal plane.

**Figure 2:**
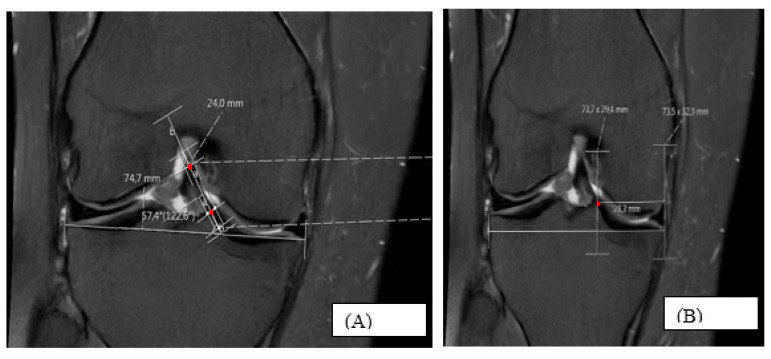
Coronal MRI measurements of the ACL tibial insertion.

In the coronal plane, the ACL coronal angle (C-ANGLE) was measured using the fibula as a lateral reference. The ACL fibers extending from the lateral femoral condyle to the tibial plateau were identified, and the femoral and tibial attachment points of the ligament were marked. A line was drawn through the midpoint between these two attachment points, and the angle between this line and the mediolateral tibial plateau was defined as the C-ANGLE [15] (Figure 2A).

For the coronal tibial footprint percentage (C-PERCENT), the first coronal slice in which the tibial attachment of the ACL was visible was selected. On this slice, the distance from the ACL tibial insertion point to the medial edge of the tibial plateau was measured as C-short, and the mediolateral length of the tibial plateau was measured as C-LONG. Perpendicular lines were drawn from the medial edge and the insertion point to the tibial plateau surface, and C-PERCENT was calculated as the ratio C-short/C-LONG expressed as a percentage [15] (Figure 2B).

(A) The coronal ACL angle (C-ANGLE) was measured on the slice identified as demonstrating the ACL in its thickest and most clearly delineated appearance. Two central midpoints were identified at separate locations along the visible ACL in this coronal section, and a line passing through these two points was drawn to define the ACL axis. The angle between this axis and the tibial plateau was calculated as the C-ANGLE. Red dots indicate the midpoint reference markers used to define the coronal ACL axis. Measurement lines correspond to PACS-generated overlays.

(B) Measurement of the coronal tibial footprint percentage (C-PERCENT), calculated as the ratio of the distance from the ACL tibial insertion to the medial tibial plateau edge (C-short) to the mediolateral tibial plateau length (C-LONG). The red dot indicates the midpoint of the ACL tibial footprint in the coronal plane. Measurement lines and numerical values represent PACS-generated measurement tools. ACL, anterior cruciate ligament; MRI, magnetic resonance imaging.

**Figure 3:**
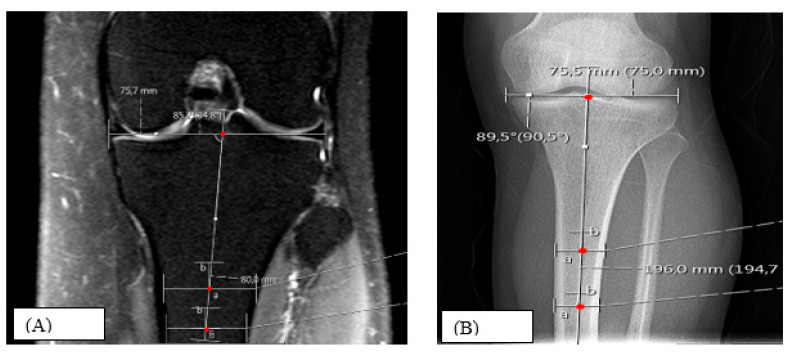
Assessment of medial mechanical proximal tibial angle (mMPTA).

To verify physiological coronal alignment and exclude marked varus or valgus deformity, the medial mechanical proximal tibial angle (mMPTA) was assessed [22]. The mMPTA was defined as the medial angle between the proximal tibial joint line and the mechanical axis of the tibia; in this study, the mechanical axis was approximated by the anatomical axis, drawn through the midpoints of the tibial diaphysis. Because the tibial shaft was not always visualized over a sufficient length on knee MRI, mMPTA measurements were performed both on coronal knee MR images and on anteroposterior (AP) knee radiographs whenever available. Cases in which a reliable mMPTA measurement could not be obtained by either method were excluded. In this study, mMPTA was used as a secondary parameter to confirm that measurements were performed on knees with physiological coronal alignment, rather than as a primary outcome variable (Figure 3).

Illustration of mMPTA measurement as the medial angle between the proximal tibial joint line and the tibial mechanical/anatomical axis on (A) coronal knee MRI and (B) anteroposterior knee radiograph, used to confirm physiological coronal alignment and exclude marked varus–valgus deformity.

mMPTA, medial mechanical proximal tibial angle; MRI, magnetic resonance imaging.

### 2.5. Observer Training, Blinding, and Reliability Analysis

To assess measurement reproducibility, a reliability analysis was performed using a randomly selected subset corresponding to 10% of the total sample (*n* = 64 knees). All measurements were independently performed by two observers experienced in musculoskeletal anatomy and MRI-based morphometric evaluation. Prior to the analysis, both observers underwent a standardized calibration session to ensure consensus regarding anatomical landmarks, reference definitions, and measurement techniques.

Observers were blinded to patient demographics, side information, and each other’s measurements. For intraobserver reliability assessment, the primary observer repeated all measurements after a washout period of at least two weeks. Interobserver and intraobserver agreement were evaluated using the intraclass correlation coefficient (ICC). The interpretation of ICC values was based on established reporting guidelines [23], according to which values <0.50 indicate poor reliability, 0.50–0.75 moderate reliability, 0.75–0.90 good reliability, and >0.90 excellent reliability.

Measurement error was quantified using the standard error of measurement (SEM) and the minimal detectable change at the 95% confidence level (MDC95). Detailed ICC values, 95% confidence intervals, SEM, and MDC95 results are presented in Table 1.

### 2.6. Statistical Analysis

Statistical analyses were performed using IBM SPSS Statistics, version 20.0 (IBM Corp., Armonk, NY, USA). The distribution of continuous variables was assessed using the Shapiro–Wilk test. Numerical variables were summarized as mean ± standard deviation and median (25th–75th percentile), while categorical variables were presented as frequencies and percentages.

Prior to study initiation, an a priori power analysis was performed to estimate the minimum required sample size. Assuming a two-sided α level of 0.05, a statistical power (1–β) of 0.99, and an effect size of 0.30, the required sample size was calculated as 207 knees. This calculation was performed during the ethical approval process. The final study sample substantially exceeded this minimum requirement, thereby providing adequate statistical power for all planned analyses and reducing the potential impact of data loss or variability.

Between-group comparisons for continuous variables that did not follow a normal distribution were performed using the Mann–Whitney U test for two-group comparisons and the Kruskal–Wallis one-way analysis of variance for comparisons involving more than two groups, followed by Dunn’s post hoc test for multiple comparisons when appropriate. Differences in categorical variables between groups were evaluated using the Pearson chi-square test and the Monte Carlo chi-square test where required. Two-sided *p* values < 0.05 were considered statistically significant.

## 3. Results

### 3.1. Demographic Characteristics

A total of 636 knees from 545 individuals were included in the analysis, comprising 338 women (53.1%) and 298 men (46.9%). Of all MRI examinations, 320 (50.3%) were obtained from the right knee and 316 (49.7%) from the left knee.

Participant age ranged from 15 to 80 years, with a mean age of 34.87 ± 11.65 years (median 33.0; 25th–75th percentile, 26.0–42.0).

For descriptive purposes, age distribution was summarized in four groups:

15–18 years: 33 knees (5.2%), mean age 16.36 ± 1.05 years
19–39 years: 397 knees (62.4%), mean age 29.41 ± 5.80 years
40–59 years: 184 knees (28.9%), mean age 46.46 ± 5.34 years
≥60 years: 22 knees (3.5%), mean age 64.50 ± 5.05 years

Detailed demographic characteristics according to sex and side are summarized in Table 2.

### 3.2. Sex-Related Differences in ACL Angles and Tibial Footprint

When the entire cohort was evaluated, significant sex-related differences were observed in ACL angular and morphometric parameters relative to the tibial plateau and anteroposterior reference-axis-based morphometric measurements (S-LONG and S-SHORT). Overall:

The sagittal ACL angle (S-ANGLE) was significantly higher in women than in men.

The anteroposterior anterior tibial reference distance (S-LONG) and mediolateral (C-LONG) dimensions of the tibial plateau, as well as the absolute distances from the ACL tibial insertion to the anterior (S-SHORT) and medial (C-SHORT) tibial margins, were significantly larger in men.

In contrast, the normalized tibial footprint percentages (S-PERCENTAGE and C-PERCENTAGE) showed only minimal differences between sexes; despite sex-related differences in tibial plateau size, the size-normalized sagittal and coronal position of the ACL tibial footprint midpoint remained within a similar range in women and men.

Median values and interquartile ranges for ACL angles and tibial footprint percentages by sex are presented in Table 3.

### 3.3. Side-to-Side Differences

Comparison of right and left knees revealed small but statistically detectable differences in some sagittal and coronal parameters. In particular, differences were noted in the mediolateral tibial plateau length and the coronal tibial footprint percentage (C-PERCENTAGE), with right knees tending to show slightly higher values.

However, the absolute magnitude of these differences was limited to a few degrees or a few percentage points, and did not substantially alter the overall angular pattern of the ACL or its size-normalized anteroposterior and mediolateral footprint position relative to the tibial plateau. Accordingly, side-to-side differences were considered secondary compared with the primary study aim of assessing sex-related morphometric variation.

Side-to-side comparisons of angle and footprint percentages are summarized in Table 4.

### 3.4. mMPTA Findings

mMPTA measurements were within the physiological coronal alignment range in the vast majority of knees. Although minor statistical differences in mMPTA values were observed among some subgroups, the absolute angular differences were not large enough to suggest clinically relevant varus or valgus deformity.

Therefore, mMPTA was used primarily as a supporting parameter to confirm that ACL measurements were obtained in knees with physiological coronal alignment; rather than as a primary morphometric outcome, detailed mMPTA values are not presented here.

### 3.5. Reliability Analysis

Interobserver and intraobserver reliability analyses demonstrated high reproducibility. Interobserver ICC values ranged from 0.830 to 0.977, whereas intraobserver ICC values ranged from 0.843 to 0.973. Based on established ICC interpretation guidelines [23], most measurements demonstrated excellent reliability, while the C-SHORT parameter showed good reliability.

Measurement error analysis revealed SEM values ranging from 0.738 to 1.524 and MDC95 values ranging from 2.045 to 4.224, indicating a low level of measurement variability relative to the observed parameter ranges. These findings confirm that the proposed morphometric measurement approach provides a high level of reproducibility with minimal observer-dependent variation. Detailed reliability metrics including ICC values, 95% confidence intervals, SEM, and MDC95 are presented in Table 1.

## 4. Discussion

### 4.1. Main Findings

In this retrospective cross-sectional MRI study of 636 knees from 545 knees without ACL injury or structural knee pathology, we provided population-specific reference values for the orientation and tibial insertion of the anterior cruciate ligament (ACL) in both the sagittal and coronal planes. Overall, the sagittal ACL angle relative to the tibial plateau (S-ANGLE) in our cohort tended to be lower than most values reported in previous MRI and cadaveric series, whereas the coronal ACL angle (C-ANGLE) showed mean values average 62.77 ± 6.32, which is close to values reported in some cadaveric studies.

A key finding of this study is that, despite men having significantly larger anteroposterior reference distances based on the anterior tibial landmark (*p* < 0.001) and mediolateral (*p* < 0.001) tibial plateau dimensions and significantly greater absolute distances from the tibial plateau margins to the ACL tibial footprint, women exhibited significantly larger S-ANGLE (*p* < 0.05) values than men. Although the difference in S-ANGLE between sexes reached statistical significance, the absolute magnitude of this difference was small (median difference approximately 0.6°). Given that multiple morphometric parameters were analyzed, this finding should be interpreted cautiously, and its clinical relevance may be limited despite statistical significance. However, when normalized for tibial plateau size, the relative anteroposterior (S-PERCENTAGE, *p* > 0.05) and mediolateral (C-PERCENTAGE, *p* > 0.05) positions of the ACL tibial insertion remained similar between genders, indicating that the ACL footprint maintains a consistent size-normalized position relative to the tibial plateau a similar relative position on the tibial plateau in both women and men, whereas overall bone geometry and ligament orientation differ. Taken together, these observations may represent an anatomical pattern that could be associated with the higher ACL injury rates reported in female populations and should be interpreted as hypothesis-generating rather than causal.

Side-to-side analyses demonstrated small but statistically detectable differences between right and left knees for some parameters (S-LONG, C-ANGLE, C-SHORT, C-LONG; *p* < 0.05); however, the absolute magnitude of these differences was small and unlikely to be clinically meaningful. The medial mechanical proximal tibial angle (mMPTA) remained within physiological limits in the vast majority of knees, supporting that the measurements were obtained in limbs with normal coronal alignment. Collectively, these findings provide a set of MRI-based morphometric reference values that may be useful for anatomical studies, radiological evaluation, and surgical planning in our population.

### 4.2. Comparison with Previous Studies

Several studies investigating the sagittal orientation of the ACL in different age groups have generally reported higher mean sagittal angle values than those observed in our cohort. In the MRI study by Kim et al. including 324 knees in individuals aged 1–20 years, the sagittal ACL angle was approximately 53° in patients with open physes and around 59° in those with closed physes, with no significant sex differences reported [4,24]. Similarly, Putur et al. showed that younger individuals tend to exhibit a more oblique ACL course in the sagittal plane. Saxena et al. reported a mean sagittal ACL angle of approximately 51° in adults with intact ligaments, Reid et al. reported 46.9° when the knee was held in a controlled flexion position during MRI, Gentili et al. found 55.6° in subjects with normal ACLs, Illingworth et al. reported 49.9° in postoperative evaluations with the knee in full extension, and Konarski et al. described a mean angle of 45.4° [14,15,25,26,27].

When these values are compared with our results, the ACL in our cohort of individuals without ACL injury or structural knee pathology appears to be oriented at a somewhat lower sagittal angle relative to the tibial plateau. Several factors may account for these discrepancies between studies:

Differences in knee flexion angle during MRI acquisition,
Variation in reference lines or axes used to define the tibial orientation (e.g., joint-line tangents, mechanical axis, or diaphyseal axis),
Differences in age distribution and body composition between populations,

Heterogeneity of study samples, including cadaveric versus in vivo imaging, pediatric versus adult cohorts, and inclusion of patients referred for arthroscopy or specific knee pathologies.

An important methodological aspect of the present study concerns the definition of the anteroposterior reference distance (S-LONG). Unlike several previous MRI studies that used the anterior margin of the tibial plateau as the primary reference, we defined S-LONG using an anterior tibial reference line derived from the midpoint of the tibial tuberosity. This approach was intentionally selected to reduce ambiguity related to the irregular contour and variable inclination of the anterior tibial plateau on sagittal MRI images. By relying on a clearly identifiable and reproducible anterior bony landmark, the resulting S-PERCENTAGE provides a size-normalized representation of the sagittal anteroposterior position of the ACL tibial footprint midpoint that is less sensitive to subtle variations in tibial plateau morphology, knee positioning, or slice orientation. Importantly, although this reference definition differs from that employed in some previous studies, the normalized footprint percentages observed in our cohort fall within the anatomic ranges reported in both MRI-based and cadaveric series, supporting the validity and cross-study comparability of this alternative reference strategy.

Thus, rather than focusing solely on absolute angle values, the main contribution of the present study lies in establishing population-specific reference range of sagittal ACL obliquity and linking these measurements to sex-related variation in size-normalized anterior tibial reference distances defined using a reproducible tibial tuberosity landmark.

With respect to the anteroposterior tibial footprint position (S-PERCENTAGE), Kim et al. reported that the relative position of the ACL tibial insertion remained around 30–31% of the tibial anteroposterior length from the anterior edge, with no significant differences between open and closed physes or between sexes. Konarski et al. reported a higher mean value of 37.3 ± 5.5% (range, 28–50%) [15]. In our cohort, normalized S-PERCENTAGE values fell within a comparable distribution, despite the use of a tibial tuberosity-based anterior reference definition, indicating that in both women and men, the ACL tibial footprint is consistently located in the middle-to-posterior region of the proximal tibia when expressed as a size-normalized percentage.

Cadaveric studies have also supported this localization [28,29]. Colombet et al. described an average distance of approximately 13 mm from the anterior tibial edge to the ACL tibial insertion and an anteroposterior tibial length of about 50 mm in adult specimens, corresponding to a similar relative position of the footprint. Despite differences in methodology, age groups, and imaging techniques, our findings are in line with these data, suggesting that the relative anteroposterior location of the ACL tibial footprint is quite robust across different populations [29].

Illingworth et al. defined an “anatomic range” for tibial tunnel placement in the sagittal plane between approximately 21% and 52% of the anteroposterior tibial length. The normalized S-PERCENTAGE values in our series largely fall within this interval, supporting the potential use of these population-based percentages as practical reference values during preoperative planning and postoperative assessment of ACL reconstructions [14].

In the mediolateral dimension, Colombet et al. reported a mean mediolateral tibial plateau length of around 78 mm in cadaveric knees and characterized the mediolateral position of the ACL footprint [29]. Putur et al., in a pediatric cohort, reported shorter mediolateral lengths (~72 mm), consistent with age-related growth differences. In our predominantly adult cohort, mediolateral dimensions were comparable to these ranges, again suggesting that our values are consistent with those reported in other anatomical and imaging studies [30].

Regarding the coronal ACL angle, several studies have reported higher mean angles than ours. Ahn et al. found a mean coronal angle of about 66° in controls and 73–75° in reconstructed knees, with no significant differences between age groups or sexes [31]. Saxena et al. reported a mean of 73.5°, whereas Reid et al. found mean values of 75.2° in women and 74.2° in men [27]. Konarski et al. reported a mean of 68.9° [15]. Conversely, Mayer et al., in a cadaveric study evaluating knees in approximately 110° flexion, reported a mean coronal ACL angle of 63.4° (range, 60.7–65.9°), which is very close to the mean values (approximately 62–63°) observed in our cohort [32]. As with sagittal angles, differences in knee flexion angle, measurement techniques, reference lines, and population characteristics likely explain much of this variation.

Importantly, consistent with the findings of Ahn, Reid, and Saxena, we did not observe a pronounced sex difference in the coronal ACL angle, which suggests that sex-related morphometric differences may be more prominent in the sagittal plane and in tibial plateau dimensions than in coronal ligament orientation [26,27,31].

### 4.3. Sex Differences, Tibial Plateau Morphology, and ACL Injury Risk

There is a substantial body of literature indicating that a steeper posterior tibial slope is associated with an increased risk of ACL injury [32,33]. In our cohort, the coronal ACL angle (C-ANGLE) showed mean values around 62–63°. Studies by Beynnon et al., Dietvorst et al., Hosseinzadeh and Kiapour, Korthaus et al., and Sutton and Bullock, among others, have shown that greater posterior tibial slope may predispose individuals to ACL rupture, particularly in high-demand and pivoting sports [17,34,35,36,37]. Although we did not directly measure tibial slope in this study, a more oblique sagittal ACL orientation in women may be functionally related to tibial slope and could represent a morphological feature that contributes to the higher reported incidence of ACL injuries in female athletes.

Epidemiological studies have consistently reported that ACL injuries occur more frequently in women than in men, especially in sports involving cutting, pivoting, and sudden deceleration [38,39]. Large clinical series by Hosseinzadeh and Kiapour, Kaeding et al., LaBella et al., Lin et al., and Wang et al. have all highlighted the increased ACL injury incidence in women [17,40,41,42,43]. The finding in our cohort that women exhibited higher S-ANGLE values and shorter anterior tibial reference distances derived from the tibial tuberosity landmark (S-LONG) compared to men is consistent with the concept that sex-related morphological and biomechanical differences in ACL geometry may contribute to increased vulnerability to injury.

Vasta et al. reported that the anteroposterior length of the tibial plateau was significantly shorter in patients with ACL rupture than in controls with intact ligaments in both sexes [44]. Interestingly, in our data, the anteroposterior tibial plateau length in women approximates the values reported for ACL-ruptured groups in Vasta’s study, whereas the values in our male participants are closer to those of the intact control group [44]. This observation supports the hypothesis that shorter tibial plateaus may be associated with increased ACL injury risk, and that women with relatively shorter plateaus could represent a structurally higher-risk subgroup [17,37,44].

Taken together, our results suggest that sex-related differences in tibial plateau size and sagittal ACL orientation may contribute to the known sex disparity in ACL injury incidence. While the present study cannot establish a causal relationship, it reinforces the concept that morphological factors should be considered alongside neuromuscular and hormonal influences when assessing ACL injury risk in women.

### 4.4. Clinical Implications for ACL Reconstruction, Tunnel Planning, and Implant Design

The anteroposterior position of the ACL tibial footprint when expressed as a size-normalized parameter (S-PERCENTAGE) is clinically relevant for tibial tunnel placement during ACL reconstruction and for postoperative MRI-based assessment. The S-PERCENTAGE values obtained in our study fall within the range reported by Kim, Konarski, Colombet, Illingworth and align with previously described anatomic placement windows, suggesting that, even when expressed using an anterior tibial reference geometry derived from the tibial tuberosity, the relative sagittal location of the ACL tibial footprint remains comparable to values reported in plateau-based measurement systems [14,15,24,29]. Accordingly, these normalized measurements may serve as practical reference thresholds for the entire reconstructive pathway, from preoperative planning of anatomical ACL reconstruction, through intraoperative evaluation of tibial guide use and tunnel positioning, to postoperative, imaging-based assessment of tunnel placement and graft orientation.

In the mediolateral plane, the mediolateral tibial plateau dimension and C-PERCENTAGE values provide complementary information regarding the coronal position of the tibial footprint. Our C-PERCENTAGE data overlap with the mediolateral “anatomic window” described by Illingworth and colleagues for tibial tunnel placement. These values can help surgeons avoid tunnels that are excessively medial or lateral, which could compromise graft isometry, rotational stability, and graft longevity.

From an engineering and design perspective, the sex-related differences in tibial plateau dimensions observed in our cohort may have implications for patient-specific instrumentation and implant sizing. Women tend to have smaller tibial plateaus and slightly different sagittal ACL orientations, which might influence the design of tibial guides, fixation devices, and even the geometry of synthetic or biological graft constructs. Integrating population- and sex-specific morphometric data into implant design could improve the anatomical compatibility of reconstruction systems, particularly in populations that may be underrepresented in current design databases.

Finally, because our measurements were performed in knees without ACL injury or advanced osteoarthritis, they provide a baseline morphometric reference dataset derived from individuals without structural knee pathology, which may help distinguish normal anatomical variability from true malposition or abnormal morphology in clinical practice. This could help distinguish between normal anatomical variability and true malposition or abnormal morphology when evaluating patients preoperatively or following ACL reconstruction.

### 4.5. Limitations and Future Directions

This study has several limitations that should be acknowledged. First, the retrospective cross-sectional design and the inclusion of patients from a single tertiary center may limit the generalizability of our findings to other populations or clinical settings. Although we excluded patients with known knee pathology and used strict inclusion and exclusion criteria, selection bias cannot be completely ruled out.

Second, MRI examinations were acquired using three different MRI systems, which may introduce subtle variability in image quality, contrast, and spatial resolution. We attempted to minimize this effect by using comparable pulse sequences and by performing all measurements on the same PACS workstation with standardized viewing conditions. Nevertheless, a fully standardized prospective imaging protocol would provide more homogeneous data. An additional methodological consideration relates to the use of multiple MRI scanners with different field strengths. Although multi-platform acquisition may theoretically introduce variability in morphometric measurements, several steps were taken to minimize this effect, including the use of standardized proton density-weighted fat-suppressed sequences, predefined anatomical reference definitions, and a uniform PACS-based measurement workflow. Importantly, the high interobserver and intraobserver ICC values observed in the present study support the robustness of the proposed measurement protocol across different imaging platforms. Rather than representing a limitation alone, the inclusion of datasets acquired from multiple scanners may enhance the external validity of the findings by reflecting real-world clinical imaging conditions.

Third, the knee flexion angle during MRI acquisition was not strictly standardized but rather reflected the maximal flexion tolerated by each patient. Since both sagittal and coronal ACL angles are known to be influenced by knee position, this variability may have contributed to the differences between our absolute angle values and those reported in other studies. However, this limitation also reflects routine clinical practice and may increase the external validity of our findings for everyday MRI interpretation. Although knee flexion angle was not standardized and reflected routine clinical positioning, this approach may increase the external validity of the findings. Future prospective studies with controlled flexion positioning may further refine angular comparisons.

Fourth, we did not directly measure posterior tibial slope or other three-dimensional alignment parameters, and we did not include patients with ACL injury. As a result, we can only infer potential associations between morphology and ACL injury risk based on comparisons with the literature, but we cannot establish a direct causal link in this cohort.

Finally, this study focused on static MRI-based measurements and did not incorporate dynamic functional assessments, such as gait analysis, muscle strength, or neuromuscular control, which are also important determinants of ACL loading and injury risk. Future studies combining 3D imaging, dynamic functional evaluations, and clinical outcomes in patients with and without ACL injury will be valuable to further clarify how the morphometric characteristics described here contribute to the complex multifactorial risk profile of ACL rupture.

Despite these limitations, the present study provides robust, population-specific reference values for ACL orientation and tibial footprint position in both the sagittal and coronal planes, and highlights sex-related differences and their potential clinical relevance for ACL injury risk and reconstruction planning.

## 5. Conclusions

In this retrospective MRI-based morphometric study of 636 knees from 545 healthy individuals, we quantified the sagittal and coronal orientation of the ACL relative to the tibial plateau and characterized its tibial footprint using size-normalized reference parameters in a large cohort from our population. To our knowledge, no previous MRI study in our country has simultaneously evaluated both angular and footprint parameters of the ACL while accounting for sex- and side-related differences in a sample of this size.

Compared with many previously published series, the ACL in our cohort exhibited a narrower range of sagittal and coronal angles relative to the tibial plateau. This discrepancy is likely multifactorial and may reflect differences in MRI acquisition (particularly knee flexion at the time of imaging), measurement techniques, anterior reference definitions used for tibial orientation and anthropometric characteristics of the studied populations rather than a single anatomical factor.

Sex-specific analyses showed that women had significantly greater sagittal ACL angles, whereas men had significantly larger anterior tibial reference distances derived from the tibial tuberosity (S-LONG) anteroposterior and mediolateral tibial plateau dimensions. Despite these size differences, normalized tibial footprint percentages (S-PERCENTAGE and C-PERCENTAGE) were broadly similar between sexes, indicating that the size-normalized relative sagittal and coronal position of the ACL tibial footprint remains preserved. However, the combination of a steeper sagittal ACL orientation and shorter anterior tibial reference geometry in women may provide a structural substrate that is consistent with the higher ACL injury rates reported in female populations.

Side-to-side comparisons demonstrated small but statistically significant differences in some anterior tibial reference-based and coronal plane parameters, with slightly higher values on the right side. Although the absolute magnitude of these differences was limited and unlikely to be clinically relevant on their own, they may be related to habitual loading patterns and limb dominance. Future studies that explicitly document and analyze limb dominance will be valuable to clarify the functional relevance of these asymmetries.

Overall, our findings provide population-specific reference values for the three-dimensional orientation and size-normalized sagittal and coronal tibial footprint position the ACL in healthy knees. These morphometric data may support preoperative planning of anatomical ACL reconstruction, MRI-based assessment of whether tibial tunnels remain within an anatomically acceptable range, and the design and refinement of ACL-related implants and devices that better respect native anatomy. In combination with future multicenter, prospective and comparative studies across different populations, the present work may contribute to a more precise understanding of ACL anatomy and to the optimization of prevention and treatment strategies for ACL injuries.

## Figures and Tables

**Table 1:** Interobserver and intraobserver reliability analysis including ICC, 95% confidence intervals, standard error of measurement (SEM), and minimal detectable change (MDC95) for all morphometric parameters.

Measurement	Type	ICC	95% CI	SEM	MDC95	*n*
C-ANGLE	Inter	0.977	0.963–0.986	1.023	2.835	64
C-ANGLE	Intra	0.970	0.952–0.982	1.141	3.163	64
C-LONG	Inter	0.973	0.956–0.984	0.980	2.717	64
C-LONG	Intra	0.969	0.950–0.981	1.051	2.913	64
C-SHORT	Inter	0.830	0.735–0.893	1.524	4.224	64
C-SHORT	Intra	0.843	0.754–0.901	1.408	3.902	64
S-ANGLE	Inter	0.965	0.943–0.979	0.831	2.305	64
S-ANGLE	Intra	0.973	0.956–0.983	0.738	2.045	64
S-LONG	Inter	0.952	0.922–0.970	1.030	2.855	64
S-LONG	Intra	0.949	0.917–0.969	1.056	2.928	64
S-SHORT	Inter	0.929	0.886–0.956	0.916	2.540	64
S-SHORT	Intra	0.933	0.892–0.959	0.927	2.570	64

**Table 2:** Demographic characteristics of the study sample according to sex and side.

Variable	*n*	%	Age, Mean ± SD (Years)
**Total**	636	100.0	34.87 ± 11.65
**Sex**			
**Women**	338	53.1	35.26 ± 11.77
**Men**	298	46.9	34.45 ± 11.52
**Side**			
**Right knee**	320	50.3	34.64 ± 11.19
**Left knee**	316	49.7	35.12 ± 12.12
**Age groups (years)**			
**15–18**	33	5.2	16.36 ± 1.05
**19–39**	397	62.4	29.41 ± 5.80
**40–59**	184	28.9	46.46 ± 5.34
**≥60**	22	3.5	64.50 ± 5.05

SD, standard deviation.

**Table 3:** Comparison of sagittal and coronal ACL angles and tibial footprint percentages between women and men.

Parameter	Women (*n* = 338) Median (25th–75th Percentile)	Men (*n* = 298) Median (25th–75th Percentile)	*p*-Value *
**S-ANGLE (°)**	37.20 (34.17–41.40)	36.60 (32.90–40.60)	0.028
**S-PERCENTAGE (%)**	40.88 (36.73–45.14)	40.74 (36.83–44.76)	0.792
**C-ANGLE (°)**	63.15 (58.67–67.00)	63.25 (59.00–66.62)	0.883
**C-PERCENTAGE (%)**	44.06 (42.60–45.35)	43.82 (42.67–45.25)	0.561

* Mann–Whitney U test. S-ANGLE, sagittal ACL angle; S-PERCENTAGE, sagittal tibial footprint percentage; C-ANGLE, coronal ACL angle; C-PERCENTAGE, coronal tibial footprint percentage.

**Table 4:** Comparison of sagittal and coronal ACL angles and tibial footprint percentages between left and right knees.

Parameter	Left Knee (*n* = 316) Median (25th–75th Percentile)	Right Knee (*n* = 320) Median (25th–75th Percentile)	*p*-Value *
**S-ANGLE (°)**	36.80 (33.62–41.40)	37.10 (33.50–40.85)	0.968
**S-PERCENTAGE (%)**	40.98 (36.01–45.25)	40.67 (37.36–44.82)	0.501
**C-ANGLE (°)**	63.15 (58.70–66.97)	63.35 (59.05–66.70)	0.633
**C-PERCENTAGE (%)**	44.03 (42.72–45.58)	43.82 (42.52–45.07)	0.122

* Mann–Whitney U test.

## Data Availability

The publicly available archive of the data analysed during the study can be accessed via this link: “https://zenodo.org/records/18326111” (accessed on 21 January 2026) and the DOI number is: https://doi.org/10.5281/zenodo.18326111.

## Abbreviations

The following abbreviations are used in this manuscript:

ACL	The anterior cruciate ligament
PCL	The posterior cruciate ligament
S-ANGLE	On sagittal images, the ACL–tibial plateau angle
S-LONG	Anteroposterior anterior tibial reference distance measured from the midpoint of the tibial tuberosity and oriented parallel to the tibial plateau
S-SHORT	Distance from the anterior tibial edge to the ACL tibial insertion
S-PERCENTAGE	S-short/S-long × 100
C-ANGLE	On coronal images, the ACL–tibial plateau angle
C-LONG	Mediolateral tibial length
C-SHORT	Distance from the medial edge to the ACL insertion
C-PERCENTAGE	C-short/C-LONG × 100
mMPTA	Medial mechanical proximal tibial angle
PACS	picture archiving and communication system
